# Effects of Hormones on Left Heart Structure and Function with Echocardiography in Acromegaly

**DOI:** 10.31083/RCM38745

**Published:** 2025-09-28

**Authors:** Haiyan Li, Rufei Shen, Hui Li, Deyu Yuan, Xiaoke Zeng, Junhui Tang, Yuling Zhang, Qinglong Li, Qiong Zhu, Xin Tan, Min Long, Yali Xu

**Affiliations:** ^1^Department of Ultrasound, Xinqiao Hospital, Army Medical University, 400037 Chongqing, China; ^2^Department of Endocrinology, Xinqiao Hospital, Army Medical University, 400037 Chongqing, China; ^3^Department of Ultrasound, The General Hospital of Tibet Military Command, 850000 Lhasa, Tibet, China; ^4^Department of Endocrinology, Southwest Hospital, Army Medical University, 400037 Chongqing, China

**Keywords:** acromegaly, growth hormone, insulin growth factor-1, cardiomyopathy, echocardiography, pituitary adenoma

## Abstract

**Background:**

The relationship between growth hormone (GH) and changes in cardiac morphology and function in the current study has not been fully elucidated, and this study aimed to assess the effect of hormonal factors on left ventricular structure and function in acromegaly patients using echocardiography.

**Methods:**

We retrospectively analyzed the relationships between various echocardiographic parameters in 117 pre-treatment patients with acromegaly and four hormonal variables: GH, GH nadir during the oral glucose tolerance test (OGTT-GH), insulin-like growth factor-1 (IGF-1), and IGF-1/upper limit of normal (IGF-1/ULN) adjusted for age and sex. Patients were categorized into normal and abnormal subgroups based on interventricular septal (IVS) thickening, left atrial (LA) enlargement, and left ventricular (LV) abnormal LV peak flow velocities E and A (E/A ratios). Furthermore, the hormonal levels within these subgroups were compared.

**Results:**

Correlation analysis revealed that IGF-1/ULN was positively associated with IVS thickening and LA enlargement (*p* = 0.003 and *p* = 0.001), and negatively associated with an abnormal LV E/A ratio (*p* < 0.001). Regression analysis identified IGF-1/ULN as a significant risk factor for left heart alterations. Among the four hormonal variables, IGF-1/ULN demonstrated the largest area under the receiver operating characteristic (ROC) curve (AUC), with values of 0.628 for IVS thickening, 0.701 for LA enlargement, and 0.653 for LV abnormal E/A ratio.

**Conclusion:**

IGF-1/ULN is strongly associated with changes in left heart structure and function in acromegaly and serves as a risk factor for these alterations. Thus, monitoring IGF-1/ULN may help predict cardiac changes via echocardiography, suggesting that early clinical management of GH-related levels could prevent early cardiac abnormalities in patients with acromegaly.

## 1. Introduction

Acromegaly, a rare chronic endocrine condition, primarily arises from growth hormone (GH) hypersecretion [1, 2, 3], with pituitary adenomas accounting for most cases [4, 5, 6, 7]. The excessive GH levels trigger increased hepatic synthesis of insulin-like growth factor-1 (IGF-1) [8]. Long-term elevated levels of GH and IGF-1 can lead to severe systemic complications, particularly affecting the cardiovascular, respiratory, and metabolic systems. These complications significantly impact the health, quality of life, and survival of patients with acromegaly [9, 10, 11]. Notably, cardiovascular complications emerge as the most significant clinical concern, contributing to a mortality rate 2–3 times higher than that of the general population [10, 12, 13]. Historically, cardiac involvement in acromegaly typically follows a progressive pattern. Early studies identified cardiac hypertrophy and fibrosis in up to 50% of acromegaly cases [13]. More recent research has shown that myocardial changes, including ventricular wall thickening and biventricular involvement (especially in the left ventricle [LV]), are common [14, 15, 16]. These structural changes frequently progress to diastolic dysfunction and impaired LV relaxation, collectively termed “acromegalic cardiomyopathy” [8, 17, 18, 19].

Advancements in medical treatments—such as surgery, pharmacotherapy, and radiation therapy—have improved the management of GH and IGF-1 levels. Normalizing these levels can reverse some of the early cardiac changes and improve heart function, particularly when IGF-1 levels are restored to normal ranges for age and sex [20, 21, 22, 23, 24]. Importantly, timely and effective treatment substantially mitigates the risk of heart failure development in affected patients [15]. These findings underscore the critical importance of early diagnosis and therapeutic intervention in acromegaly management.

Echocardiography plays an essential role in diagnosing cardiac abnormalities, as it can assess ventricular wall thickness, atrioventricular size, and both systolic and diastolic function [25, 26]. While the relationship between GH and cardiac morphological changes is still under investigation, this study aims to clarify the impact of long-term hormonal stimulation on heart structure and function. By analyzing echocardiographic parameters and clinical data from 117 untreated acromegaly patients, we aim to identify factors influencing cardiac changes and provide a basis for early intervention, potentially improving cardiac outcomes and reducing the incidence of long-term cardiac complications in these patients [1].

## 2. Methods

### 2.1 Participants

A total of 117 patients diagnosed with GH-secreting pituitary adenoma and admitted to our hospital between January 2016 and August 2022 were selected for the study. The diagnosis of acromegaly was made based on the criteria established by the Chinese Consensus on the Diagnosis and Treatment of Acromegaly (2021 Edition): (1) typical clinical manifestations of acromegaly, including facial changes, hand and foot hypertrophy, and tongue enlargement [1]; (2) a GH nadir during the oral glucose tolerance test (OGTT-GH) ≥1 µg/L; (3) IGF-1 levels exceeding the upper limit of normal (ULN) for age and sex; and (4) magnetic resonance imaging (MRI) showing evidence of a pituitary tumor. Patients who did not meet these criteria or who had congenital heart disease, severe valvular disease (e.g., aortic stenosis, aortic regurgitation, and mitral regurgitation, etc.) [27, 28, 29], or cardiomyopathy were excluded.

A control group consisting of 117 patients with non-functioning pituitary adenomas, matched for gender and age, was also selected. Inclusion criteria for the control group were: (1) MRI evidence of a pituitary adenoma; (2) normal hormone levels without signs or symptoms of hormone hyperactivity. Patients with congenital heart disease or severe valvular disease were excluded from the control group as well.

The study protocol was approved by the Human Research Ethics Committee of Xinqiao Hospital (No. 2021-035-02). As no personally identifiable information was used in this study, informed consent from individual participants was not required.

### 2.2 Clinical Data Collection

Demographic data collected from both groups included sex, age, height, weight, body mass index, body surface area, and disease duration. Laboratory biochemical indicators measured included GH-related parameters such as random GH levels, IGF-1, and OGTT-GH. The IGF-1/ULN ratio was calculated by comparing the measured IGF-1 levels to the ULN for age and sex. The levels of GH were measured using a double-antibody sandwich enzyme-linked immunosorbent assay (ELISA) with the Human GH ELISA Kit from Redit Bio-Tech (Wuhan) Co., Ltd, Catalog NO.: RE1087H.

### 2.3 Echocardiographic

#### 2.3.1 Examination and Views

In this study, we employed a variety of echocardiography devices, including the Philips IE33 (Philips Ultrasound, Inc, Bothell, Washington, USA), ELITE (Philips Ultrasound, Inc, Bothell, Washington, USA), EQ7 (Philips Healthcare (Suzhou) Co., Ltd, Suzhou City, Jiangsu Province, China), and GE VIVID E9 and VIVID E95 (GE Vingmed Ultrasound AS, Horten, Norway). During the examinations, phased-array ultrasound probes were utilized, with frequency ranges adjusted according to the specific device and patient condition to optimize image quality. Standard views obtained during echocardiography included the parasternal long-axis view (PLAX) and the parasternal short-axis view (PSAX). These views facilitated systematic measurements of cardiac chamber dimensions, great vessel diameters, and the assessment of cardiac function. All measurements were strictly conducted in accordance with standardized echocardiography guidelines to ensure data accuracy and reproducibility [26]. The echocardiographic data were analyzed by experienced cardiac sonographers and meticulously documented in the electronic medical record system. During the data analysis process, key indicators were repeatedly verified to ensure data accuracy and consistency.

#### 2.3.2 Measurement

Echocardiographic parameters were assessed according to the methods recommended by the American Society of Echocardiography and the European Society of Cardiovascular Imaging. These included measurements of the interventricular septal thickness (IVST), LV posterior wall thickness (LVPWT), LV end-diastolic diameter (LVDd), left atrial (LA) anteroposterior diameter (LAD), right atrial transverse diameter (RAD), right ventricular transverse diameter (RVD), aortic root diameter (AORD), ascending aortic diameter (AAOD), main pulmonary artery diameter (PAD), and LV peak flow velocities (E and A) at the early and late stages of mitral valve diastolic filling. Stroke volume (SV), LV ejection fraction (LVEF), and the E/A ratio were also calculated. According to Chinese cardiac ultrasound guidelines, normal values for echocardiographic parameters are as follows: LAD <37 mm for women and <39 mm for men, LVDd <50 mm for women and <55 mm for men, RVD/RAD <40 mm, and IVST <11.5 mm, with a normal LVEF range of 55–75% and a normal E/A ratio between 0.8 and 2.0. LV systolic function was evaluated by measuring systolic and diastolic diameters and ventricular wall thickness using M-mode and 2D echocardiography, with LVEF calculated using geometric formulas [30]. LV diastolic function was assessed based on the early and late peak flow velocity E/A ratio obtained from the apical four-chamber view [31].

### 2.4 Calculation of the Cardiac Index

The LV mass (LVM), LVM index (LVMI), and relative wall thickness (RWT) were calculated using the Devereux formula. LVMI was calculated as LVM/BSA, with the reference ULN LVMI set at 95 g/m^2^ for women and 115 g/m^2^ for men. An LVMI exceeding these reference values was considered indicative of LV hypertrophy (LVH) [26]. RWT and LVH were used to further assess cardiac geometry. If LVH was present, an RWT ≤0.42 cm was considered indicative of eccentric hypertrophy (EH), while an RWT >0.42 cm suggested concentric hypertrophy (CH). If LVH was absent, an RWT >0.42 cm indicated concentric remodeling (CR) of the LV [13].

### 2.5 Statistical Analysis

Data were analyzed using SPSS software (version 26; IBM, Armonk, NY, USA) and R-Studio software (version 2023.09.1 + 494; PBC Boston, MA, USA). Kolmogorov–Smirnov normality test was used to test the normality of continuous variables. Continuous variables that were normally distributed or approximately normal were presented as mean ± standard deviation. For normally distributed variables, comparisons between two groups were made using the independent samples *t*-test, while comparisons among three groups were performed using analysis of variance (ANOVA). For continuous variables with severely skewed distributions, data were presented as median and interquartile range *M (P_25_, P_75_)*, and comparisons between two groups were conducted using the Mann-Whitney U test. Comparisons among three groups were made using the Kruskal-Wallis H test. Categorical variables were expressed as n (%), and the chi-square test was used to compare frequencies between groups. Correlations between variables were performed using the Pearson correlation test or Spearman’s rank correlation analysis. Logistic regression analysis was applied to investigate the relationship between cardiac parameters and potential influencing factors. Diagnostic values were assessed using sensitivity, specificity, and the area under the receiver operating characteristic curve (AUC-ROC). ROC curves derived from multivariate analysis were used to predict the association between IGF-1/ULN and the risk of developing structural and functional cardiac changes. All statistical tests were two-sided, and a *p*-value of <0.05 was considered statistically significant.

## 3. Results

### 3.1 Comparison of General Information and Echocardiographic Parameters Between the Groups

There were no significant differences in general demographic data between the acromegaly group and the control group. However, echocardiographic measurements, including the IVST, LVPWT, LAD, LVDd, RAD, RVD, PAD, AORD, AAOD, and SV, were all significantly higher in the acromegaly group than in the control group. Additionally, the prevalence of LV abnormal LV peak flow velocities E and A (E/A ratios) was significantly higher in the acromegaly group (*p* < 0.05). No significant differences were observed between the groups in LVEF or the E and A velocities (Table 1).

**Table 1.** **General information and echocardiographic parameters of patients in the acromegaly and control groups**.

Variables		Acromegaly (n = 117)	Control (n = 117)	*t/Z/χ^2^*	*p*
General information					
	Gender (F:M)	68:49	65:52	0.157	0.692
	Age (years)	45.79 ± 11.96	47.08 ± 12.39	0.805	0.421
	Duration of disease (years)	4.00 (1.00, 9.50)	0.50 (0.08, 1.00)	8.011	<0.001***
	Height (m)	1.64 ± 0.08	1.61 ± 0.08	2.631	0.009**
	Weight (Kg)	70.07 ± 10.88	65.20 ± 10.94	3.409	0.001**
	BSA (m^2^)	1.72 ± 0.17	0.67 ± 0.14	51.304	<0.001***
	BMI (Kg/m^2^)	25.95 ± 3.08	25.01 ± 3.73	2.116	0.035*
	GH (µg/L)	19.30 (9.94, 50.90)	0.84 (0.26, 1.69)	12.957	<0.001***
	IGF-1 (µg/L)	726.60 ± 233.04	137.43 ± 65.39	26.329	<0.001***
	IGF-1/ULN	2.79 ± 0.92	0.52 ± 0.23	25.826	<0.001***
Echocardiographic parameters					
	IVST (mm)	11.0 (10.0, 12.0)	10.0 (10.0, 11.0)	4.839	<0.001***
	LVPWT (mm)	10.0 (10.0, 10.9)	10.0 (9.0, 10.0)	4.709	<0.001***
	LAD (mm)	33.87 ± 3.85	31.49 ± 2.71	5.470	<0.001***
	LVDd (mm)	46.18 ± 3.59	43.99 ± 2.94	5.104	<0.001***
	RAD (mm)	35.0 (34.0, 37.0)	33.0 (31.0, 35.0)	6.020	<0.001***
	RVD (mm)	34.0 (33.0, 36.0)	32.0 (30.0, 34.0)	5.922	<0.001***
	PAD (mm)	23.0 (22.0, 24.0)	21.0 (20.0, 22.0)	4.941	<0.001***
	AORD (mm)	32.58 ± 3.16	30.98 ± 2.82	4.107	<0.001***
	AAOD (mm)	31.14 ± 2.74	30.11 ± 2.52	2.982	0.003**
	LVEF (%)	65.49 ± 5.14	66.18 ± 4.43	1.104	0.271
	SV (mL)	71.00 (61.00, 81.50)	62.00 (52.50, 78.00)	3.609	<0.001***
	E (cm/s)	72 (58, 85)	77 (63, 87)	1.568	0.117
	A (cm/s)	81 (64, 92)	74 (63, 86)	1.656	0.098
	Abnormal E/A ratio (n%)	51 (43.6%)	31 (26.5%)	7.510	0.006**
	LVM (g)	175.66 ± 35.51	147.26 ± 25.82	6.997	<0.001***
	LVMI (g/m^2^)	102.31 ± 20.40	225.62 ± 51.40	24.117	<0.001***
	RWT (cm)	0.46 ± 0.06	0.45 ± 0.04	1.672	0.096

Notes: Data are expressed as mean ± standard deviation, *M (P_25_, P_75_)*, or percentage. *p*-values were obtained using an independent samples *t*-test, Mann-Whitney U test, or chi-square test. Abbreviations: GH, growth hormone; IGF-1, insulin-like growth factor-1; BSA, body surface area; BMI, body mass index; IGF-1/ULN, ratio of IGF-1 to age- and sex-matched IGF-1 upper normal limit; IVST, end-diastolic interventricular septal thickness; LV, left ventricular; LVPWT, left ventricular posterior wall thickness; LAD, left atrial anteroposterior diameter; LVDd, left ventricular end-diastolic diameter; RAD, right atrial transverse diameter; RVD, right ventricular transverse diameter; AORD, aortic root diameter; AAOD, ascending aortic diameter; PAD, pulmonary artery diameter; LVEF, left ventricular ejection fraction; SV, stroke volume; E, early peak diastolic filling velocity at the mitral valve; A, late peak diastolic filling velocity at the mitral valve; abnormal E/A ratio, abnormal E/A ratio at the mitral valve; LVM, left ventricular mass; RWT, relative wall thickness; LVMI, LVM index; RWT, relative wall thickness. *p < 0.05, **p < 0.01, ***p < 0.001.

**Fig. 1.**
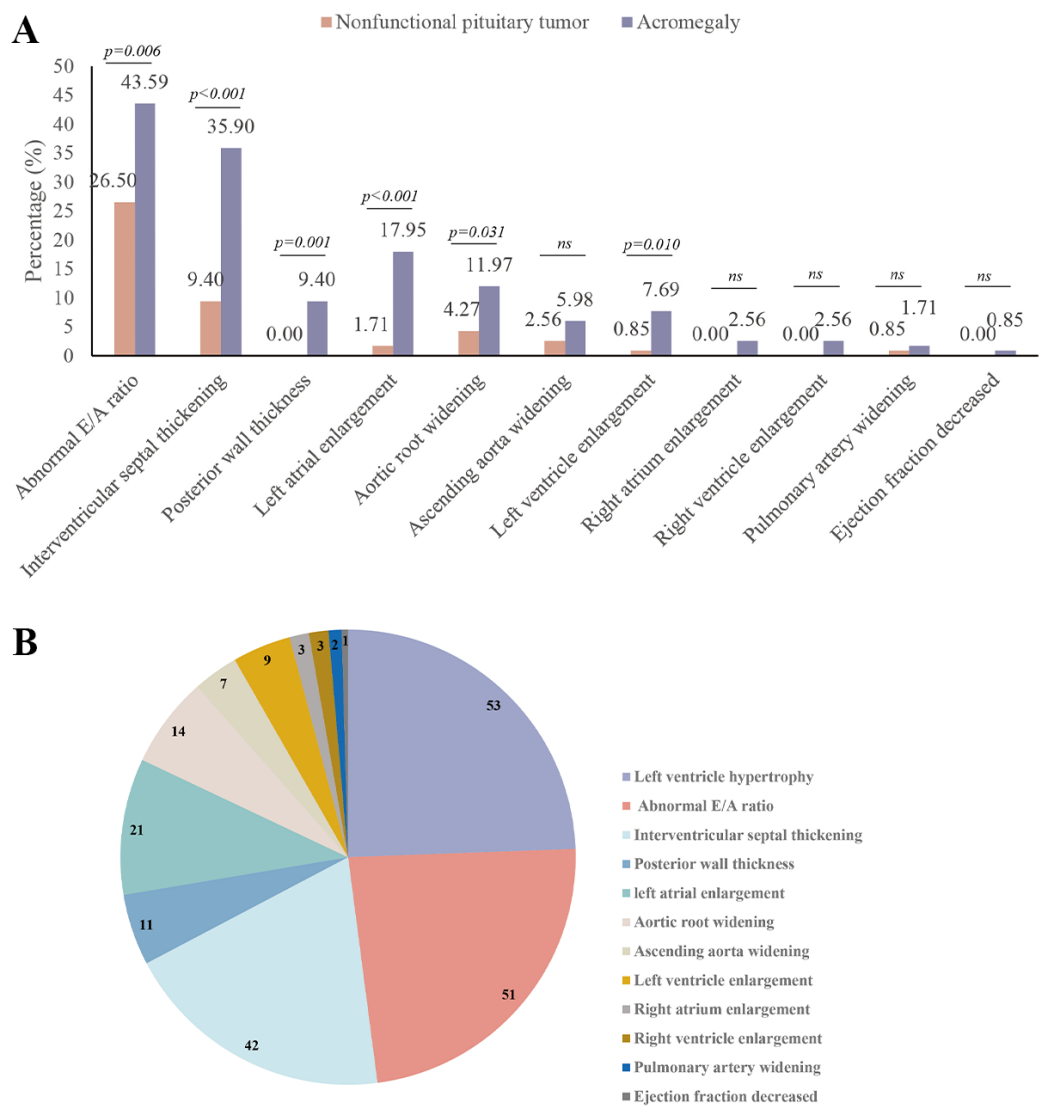
**Percentage and number of abnormal echocardiographic parameters in the acromegaly Group**. (A) Percentage of various characteristic cardiac changes in the acromegaly and control groups. (B) Number of cases with various heart structural and functional abnormalities.

The IVST, LVPWT, LAD, and LVDd were significantly greater in the acromegaly group than in the control group, and the proportion of individuals with these cardiac changes was also higher (Fig. 1A). The LVM in the acromegaly group (175.66 ± 35.51) g was significantly higher than that in the control group (147.26 ± 25.82) g, with 53 cases (45.3%) of LVH observed in the acromegaly group (Fig. 1B) [Among these, 39 cases (33.3%) exhibited CH (RWT >0.42 cm), and 14 cases (12.0%) showed EH (RWT ≤0.42 cm)]. Additionally, 49 cases (41.9%) of LV CR were noted.

### 3.2 Correlation Analysis of Echocardiographic Parameters in the Acromegaly Group

To further explore the relationship between echocardiographic parameters and GH-related factors, correlation analysis was conducted. The results revealed that the IGF-1/ULN ratio was positively correlated with LVM, IVST, LAD, and A velocity, and negatively correlated with E velocity and the E/A ratio. No significant correlations were found between random GH, OGTT-GH, or IGF-1 levels and echocardiographic parameters (Table 2).

**Table 2.** **Correlation between echocardiographic and GH-related parameters**.

Variables	GH		IGF-1		IGF-1/ULN		OGTT-GH	
	r	*p*	r	*p*	r	*p*	r	*p*
LVM^a^	–0.013	0.888	0.130	0.163	0.221	0.017*	–0.052	0.576
LVMI^a^	–0.024	0.801	–0.028	0.768	0.055	0.554	–0.065	0.485
RWT^a^	–0.073	0.436	0.075	0.424	0.212	0.022*	–0.042	0.656
IVST^b^	–0.064	0.492	0.113	0.227	0.270	0.003**	–0.090	0.333
LVPWT^b^	–0.028	0.763	0.112	0.190	0.215	0.020*	0.003	0.971
LAD^a^	0.124	0.182	0.125	0.181	0.301	0.001**	0.077	0.411
LVDd^a^	0.048	0.604	0.054	0.653	0.027	0.776	–0.010	0.915
AORD^a^	0.027	0.776	0.005	0.960	0.086	0.354	–0.046	0.619
AAOD^a^	0.010	0.918	0.093	0.320	0.208	0.024*	–0.046	0.624
EF^a^	–0.198	0.032*	0.064	0.492	0.046	0.625	–0.167	0.072
SV^b^	0.068	0.466	0.036	0.698	0.125	0.179	0.049	0.601
E^b^	0.066	0.481	–0.044	0.637	–0.190	0.040*	0.036	0.698
A^b^	–0.028	0.768	–0.023	0.805	0.285	0.002**	0.055	0.553
E/A ratio^a^	0.109	0.244	–0.009	0.925	–0.332	0.000***	–0.002	0.982

Notes: Correlations between variables were analyzed using Pearson’s correlation analysis or Spearman’s rank correlation analysis. ^a^ Pearson correlation analysis was used for variables that conform to a normal distribution. ^b^ Spearman rank correlation analysis was used for variables that did not conform to a normal distribution. Abbreviations: OGTT-GH, GH nadir during oral glucose tolerance test; IGF-1/ULN, ratio of IGF-1 to age- and sex-matched IGF-1 upper normal limit. *p < 0.05, **p < 0.01, ***p < 0.001.

### 3.3 Subgroup Analysis of Different Cardiac Abnormalities

Based on the correlation analysis results (Table 2) and the percentage of cases with abnormal echocardiographic parameters (Fig. 1), the acromegaly group was subdivided into normal and abnormal subgroups according to the presence of interventricular septal (IVS) thickening, LA enlargement, and abnormal LV E/A ratios. The differences in hormone levels among these subgroups were further analyzed (Table 3), and logistic regression was used to assess the risk factors for structural and functional abnormalities of the left heart (Table 4).

**Table 3.** **Comparison of cardiac changes between the normal and abnormal groups**.

Variables		Abnormal group	Normal group	*t/Z*	*p*
Interventricular septal thickening					
	Number	42	75		
	OGTT-GH (µg/L)	11.15 (5.71, 23.43)	15.10 (5.85, 39.10)	1.253	0.210
	GH (µg/L)	15.80 (9.47, 29.58)	21.80 (10.50, 58.90)	1.318	0.187
	IGF-1 (µg/L)	750.05 ± 246.12	713.47 ± 226.02	0.813	0.418
	IGF-1/ULN	3.04 ± 0.98	2.65 ± 0.86	2.232	0.028*
Left atrial enlargement					
	Number	21	96		
	OGTT-GH (µg/L)	18.20 (8.08, 65.74)	12.10 (5.35, 34.95)	1.449	0.147
	GH (µg/L)	23.00 (10.35, 83.50)	19.20 (9.85, 39.95)	0.590	0.556
	IGF-1 (µg/L)	788.33 ± 228.57	713.09 ± 233.00	1.345	0.181
	IGF-1/ULN	3.33 ± 1.00	2.67 ± 0.86	3.100	0.002**
Abnormal E/A ratio					
	Number	51	66		
	OGTT-GH (µg/L)	13.20 (6.75, 47.90)	13.90 (4.85, 35.10)	0.838	0.402
	GH (µg/L)	21.00 (10.20, 35.30)	19.00 (10.08, 54.05)	0.118	0.906
	IGF-1 (µg/L)	733.84 ± 210.69	721.00 ± 250.40	0.294	0.769
	IGF-1/ULN	3.06 ± 0.91	2.57 ± 0.87	2.955	0.004**

Notes: Data are expressed as mean ± standard deviation, *M (P_25_, P_75_)*, and analyzed using an independent samples *t*-test or Mann-Whitney U test. *p < 0.05, **p < 0.01.

**Table 4.** **Logistic regression analysis of the three subgroups**.

Multivariate analysis							
Variables		b	S_b_	Wald χ^2^	*p*	OR	95% CI
Interventricular septal thickening							
	OGTT-GH	–0.015	0.011	1.799	0.180	0.986	0.965–1.017
	GH	0.002	0.007	0.106	0.745	1.002	0.989–1.015
	IGF-1	–0.004	0.002	3.776	0.052	0.996	0.992–1.000
	IGF-1/ULN	1.480	0.533	7.697	0.006**	4.393	1.544–12.496
Left atrial enlargement							
	OGTT-GH	0.006	0.014	0.208	0.649	1.006	0.980–1.034
	GH	–0.002	0.011	0.034	0.853	0.998	0.976–1.020
	IGF-1	–0.008	0.003	6.271	0.012*	0.992	0.986–0.998
	IGF-1/ULN	2.449	0.752	10.608	0.001**	11.576	2.652–50.535
Abnormal E/A ratio							
	OGTT-GH	0.027	0.014	3.548	0.060	1.027	0.999–1.057
	GH	–0.015	0.011	1.787	0.181	0.985	0.965–1.007
	IGF-1	–0.014	0.003	18.175	<0.001***	0.986	0.980–0.993
	IGF-1/ULN	–1.205	0.737	2.676	<0.001***	44.424	8.756–225.400

Note: Results of binary logistic regression analyses are presented. Abbreviations: OR, odds ratio; CI, confidence interval. *p < 0.05, **p < 0.01, ***p < 0.001.

#### 3.3.1 Comparison of GH-Related Between the Normal and Abnormal Groups

Comparison results from the three subgroups showed that IGF-1/ULN was significantly higher in the abnormal groups with IVS thickening, LA enlargement, and LV abnormal E/A ratio than in the normal groups (3.04 ± 0.98 vs. 2.65 ± 0.86, 3.33 ± 1.00 vs. 2.67 ± 0.86, and 3.06 ± 0.91 vs. 2.57 ± 0.87, respectively). These differences in IGF-1/ULN were statistically significant between the abnormal and normal groups (Table 3). Although no statistically significant differences were found in GH levels between the normal and abnormal subgroups, GH levels in the abnormal E/A ratio group and the LA enlargement group were higher than those in the normal groups. No significant differences in GH, OGTT-GH, IGF-1, or IGF-1/ULN were observed across the three abnormal subgroups (**Supplementary Table 1**).

#### 3.3.2 Logistic Regression Analysis of the Abnormal Groups

The logistic regression analysis for screening risk factors for structural and functional abnormalities in acromegalic cardiomyopathy revealed that IGF-1/ULN was significantly associated with IVS thickening (OR: 4.393, 95% confidence interval (CI): 1.544–12.496, *p* = 0.006), LA enlargement (OR: 11.576, 95% CI: 2.652–50.535, *p* = 0.001), and LV abnormal E/A ratio (OR: 44.424, 95% CI: 8.756–225.400, *p* < 0.001) (Table 4). These findings suggest that IGF-1/ULN could be an independent risk factor for abnormal cardiac changes.

### 3.4 Analysis of the Clinical Significance of GH-Related Hormones in Various Subgroups With Left Heart Changes

**Fig. 2.**
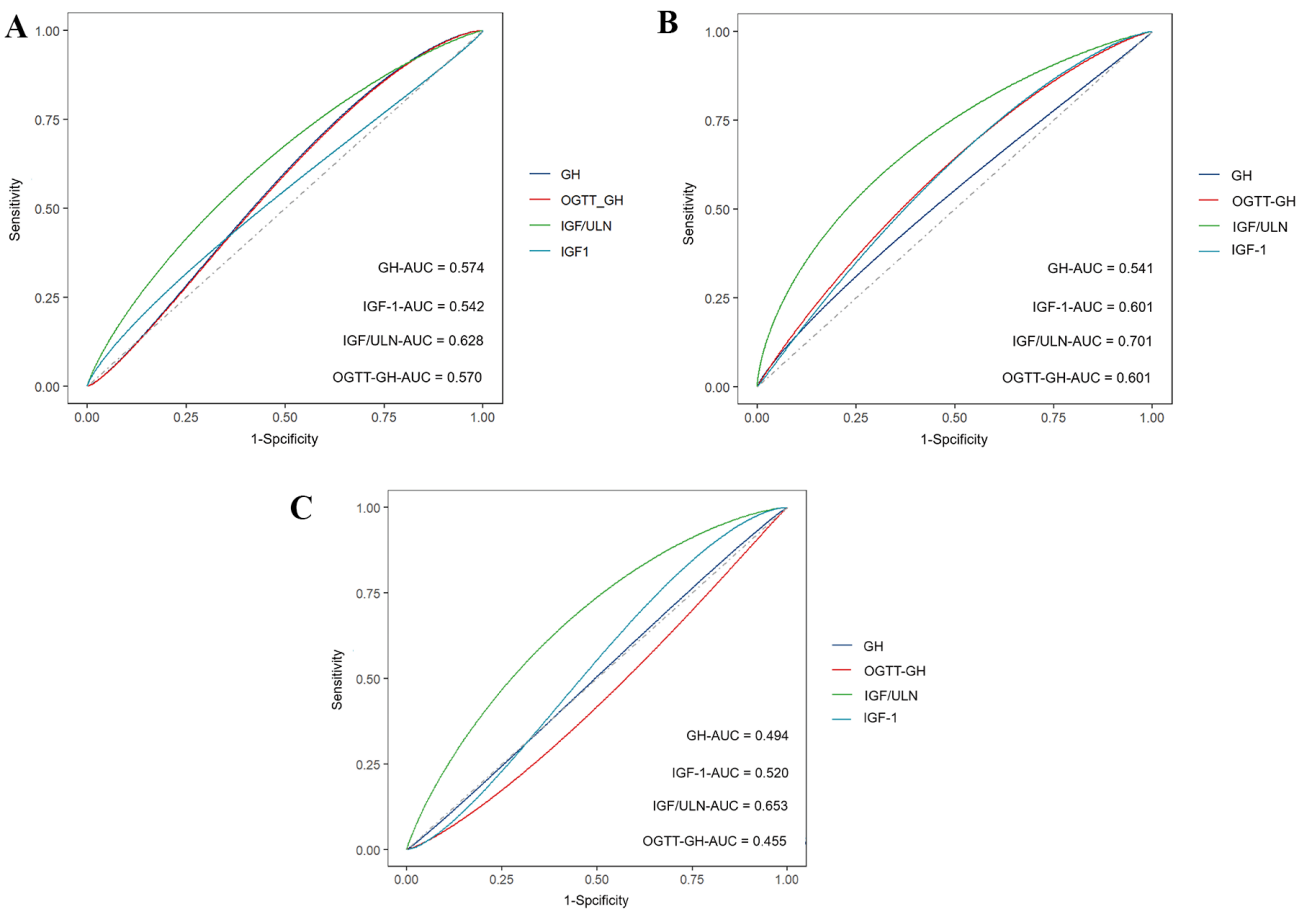
**Receiver operating characteristic curves of GH, OGTT-GH, IGF-1, and IGF-1/ULN**. (A) ROC curve for IVS thickening, (B) ROC curve for LA enlargement, and (C) ROC curve for abnormal E/A ratio. Notes: GH, growth hormone; IGF-1, insulin-like growth factor-1; IGF-1/ULN, ratio of IGF-1 to age- and sex-matched IGF-1 upper normal limit; ROC, receiver operating characteristic; OGTT-GH, GH nadir during the oral glucose tolerance test; AUC, the area under the receiver operating characteristic curve.

ROC curves were used to assess the diagnostic value of GH, OGTT-GH, IGF-1, and IGF-1/ULN in relation to left heart changes in the three subgroups. AUC values (Fig. 2), along with the corresponding cutoff values, sensitivities, and specificities, are presented in **Supplementary Table 2**. The analysis showed that among the four variables, only the IGF-1/ULN value was statistically significant (*p* < 0.05). Specifically, patients with an IGF-1/ULN greater than 3.43 before treatment were more likely to exhibit IVS thickening on echocardiography (AUC: 0.628, 95% CI: 0.324–0.536, *p* = 0.023) (Fig. 2A), those with an IGF-1/ULN greater than 3.03 were more likely to show LA enlargement (AUC: 0.701, 95% CI: 0.575–0.826, *p* = 0.004) (Fig. 2B), and those with an IGF-1/ULN greater than 2.49 were more likely to have an abnormal LV E/A ratio (AUC: 0.653, 95% CI: 0.555–0.753, *p* = 0.004) (Fig. 2C). These findings suggest that the degree of IGF-1/ULN could serve as a useful predictor of structural and functional changes in the left heart in acromegalic patients.

## 4. Discussion

This retrospective study used echocardiography to evaluate cardiac morphology and function in patients with acromegaly. We found that compared with controls, patients with acromegaly exhibited increased LV chamber diameter, wall thickening, LVM, and a higher prevalence of abnormal E/A ratios. These cardiac changes were associated with IGF-1/ULN levels and may serve as risk factors. Early control of these hormone levels is critical for preventing or treating cardiac structural and functional changes in acromegaly. Echocardiography, a widely used clinical tool, enables real-time monitoring of cardiac changes, providing valuable imaging for early detection of structural and functional alterations, as well as guidance for appropriate treatment and monitoring of cardiac reversal after IGF-1 normalization.

LV wall thickening, LA enlargement, and LV diastolic dysfunction were the most common cardiac changes observed in acromegalic patients, occurring in 35.9%, 17.95%, and 43.59% of cases, respectively. In contrast, fewer than 1% of patients exhibited reduced systolic function, and only 2.5% had biventricular enlargement. These findings are consistent with those of Popielarz-Grygalewicz *et al*. [4], who described the stages of acromegaly cardiomyopathy: early (asymptomatic LVH and increased systolic output), middle (significant LVH during exercise, diastolic dysfunction, and decreased systolic output), and late (ventricular dilation and systolic dysfunction). Notably, some studies have confirmed that systolic dysfunction and ventricular dilatation predominantly manifest during advanced disease stages [16, 32]. A study on rats shows that pathological induction of LVH will increase the risk of cardiac events and arrhythmia, and even lead to heart failure [33]. According to another report on 8 acromegaly patients, LVH and fibrosis may be the cause of arrhythmia development [34]. Therefore, early attention to the occurrence of LV enlargement and diastolic dysfunction by echocardiography is helpful to prevent further arrhythmia in the clinic.

Chen *et al*. [35] reported that 24 (60%) of 40 patients with acromegaly had IVS thickening, and believed that IGF-1 levels were positively correlated with the risk of IVS thickening. Correspondingly, Baykan *et al*. [36] reported positive correlations between GH excess and increased IVST. In contrast to these findings, our analysis revealed several distinct observations. First, patients exhibiting abnormal IVS thickening displayed significantly higher IGF-1/ULN levels compared to controls, which may be a risk factor for IVS thickening. Importantly, we did not observe a significant correlation with GH or IGF-1 levels themselves. Second, the rate of IVS thickening in our study (35.9%) was lower than the 60% reported by Chen *et al*. [35]. This discrepancy may be due to differences in study design, previous studies have shown that the conclusion that elevated IGF-1 increases the risk of IVS thickening is stable and reliable, but IGF-1 secretion decreases with increasing age, so the threshold of IGF-1 varies at different ages, and the threshold of gender is also different, so the accuracy of IGF-1 analysis without gender and age correction will decrease. Such as Yuan Chen’s study [35] did not adjust for age and gender in the analysis of IGF-1 levels, and the sample size was relatively small (40 cases). Our study adjusted IGF-1 for age and gender, and included a larger sample size, which likely reduced potential biases. Previous studies have suggested that among factors such as age, gender, BMI, GH, and IGF-1 concentration, IGF-1 is the most influential factor in acromegalic cardiac changes, though it has not always reached statistical significance [4, 37]. This may be due to the lack of detailed subgroup analysis in some studies, whereas our study focused on the specific subgroup of IVS thickening, which may have heightened the sensitivity of the correlations between cardiac structural changes and hormone levels.

Our study also found that IGF-1/ULN could be a risk factor for LA enlargement. The LAD was significantly larger in acromegalic patients than in controls, and IGF-1/ULN levels were notably higher in the LA enlargement group. Correlation and regression analyses also indicated that LA enlargement had a strong relationship with IGF-1/ULN, suggesting that it may be a key risk factor. This finding contrasts with the study by Popielarz-Grygalewicz *et al*. [4]. It was reported that their study showed LA enlargement in 62% of 140 patients, a proportion significantly higher than the 17.95% observed in our study. They attributed LA enlargement to LV hypertrophy, impaired diastolic function, and increased GH receptor expression in LA cardiomyocytes. In contrast, our study suggests that LA enlargement may be primarily related to elevated IGF-1 levels, as GH effects on the heart are largely mediated through IGF-1 [13, 38, 39]. IGF-1 induces myocardial cell hypertrophy by upregulating myosin light chain 2, troponin T type 2, and skeletal muscle α-actin without altering cardiac α-actin expression, while exerting acute inotropic effects via increased intracellular calcium and anti-apoptotic effects on myocardium [12, 40]. However, sustained excessive hormone stimulation leads to cardiac morphological changes and dysfunction, which may lead to impaired myocardial relaxation and increased wall thickness in the early stage, and serious heart failure in the late stage [9]. Additionally, for the first time, we performed subgroup analyses in acromegalic patients based on abnormal ultrasound parameters, providing a more detailed investigation of the relationship between abnormal ultrasound findings and related hormones. It is important to note that LA enlargement has been linked to an increased risk of arrhythmias, particularly atrial fibrillation [4]. Therefore, clinicians should monitor IGF-1 levels before and after treatment, alongside regular echocardiographic follow-ups to assess LA size.

IGF-1/ULN may represent a critical biomarker for predicting cardiac structural and functional alterations in acromegaly. Recent investigations have primarily evaluated GH and IGF-1 levels as predictors of long-term therapeutic outcomes and comorbidities. For instance, a meta-analysis found that IGF-1 levels were the most reliable predictor of biochemical response to first-generation somatostatin analog therapy [10, 18]. However, studies specifically examining hormones as predictors of cardiac changes remain scarce. Our clinical observations and statistical analyses suggest that LV diastolic dysfunction may be the first detectable change in acromegalic patients, as nearly half of the patients in our study exhibited this dysfunction. When IGF-1/ULN exceeded 2.49, the probability of an abnormal E/A ratio increased. As IGF-1/ULN reached levels of 3.03 and 3.49, LA enlargement and IVS thickening were more likely to occur. These observations corroborate existing evidence that LA dilation may stem from early diastolic impairment, with subsequent volume overload exacerbating ventricular hypertrophy [4]. Collectively, our findings position IGF-1/ULN as both a predictive marker for cardiac abnormalities and a potential risk stratification tool in acromegaly management. Future research directions include longitudinal assessment of treatment-induced cardiac function changes and the development of predictive models for cardiac damage reversibility. This study is the first to conduct a subgroup analysis of acromegalic patients based on individual echocardiographic abnormalities. By directly comparing hormone levels between normal and abnormal groups, we were able to identify influencing factors and correlations.

However, the study has some limitations. First, its retrospective design limits the ability to establish causality, because this kind of research relies on the data collected in the past, making it difficult to fully control and adjust all potential confounding factors. Additionally, the heart is a complex geometry, the use of conventional echocardiography, which is limited by its angle dependence and the single-plane data collection, and it is difficult to measure its volume and function accurately by 2D echocardiography, which may affect the accuracy of cardiac function evaluation and lead to underestimation or overestimation of left ventricular function, thus underestimating subclinical dysfunction and judging the severity and prognosis of the disease.

Recent advancements in speckle tracking echocardiography (STE) can overcome the angle dependence of traditional 2D grayscale ultrasound, allowing for multi-sectional tracking and more accurate measurements of myocardial displacement and strain. STE is particularly useful for assessing global longitudinal strain and myocardial work parameters, and LV pressure-strain loops (PSL) combined with STE reflect regional myocardial work [41], which are sensitive indicators of myocardial function. STE and PSL could enhance the early detection of subclinical LV systolic dysfunction and diastolic impairment, providing more accurate assessments of cardiac function [31, 42, 43]. In our future studies, combining STE with regular monitoring of cardiac strain values before and after treatment will help evaluate early cardiac dysfunction and the reversal of myocardial damage. Additionally, integrating clinical data and hormone levels to develop predictive models for early cardiac damage and its subsequent reversal could provide a reliable imaging basis for diagnosing and treating acromegalic cardiomyopathy at various stages. Early intervention may help delay cardiac structural changes, improve cardiac function, and reduce the incidence of adverse events and hospital readmissions related to cardiovascular disease.

## 5. Conclusions

This study demonstrates that changes in left heart structure and function in acromegalic patients are not directly associated with GH levels, but are correlated with IGF-1/ULN, which serves as a risk factor. When IGF-1/ULN reaches a certain threshold, LV diastolic dysfunction is often the first detectable echocardiographic change. Early clinical control of relevant hormone levels is essential to prevent the progression of LV diastolic dysfunction and to mitigate or slow the development of irreversible myocardial damage.

## Availability of Data and Materials

The data that support the findings of this study are available from the corresponding author upon reasonable request.

## Supplementary Material

Supplementary material associated with this article can be found, in the online version, at https://doi.org/10.31083/RCM38745.
