# Supplementary material for: Effects of Hormones on Left Heart Structure and Function with Echocardiography in Acromegaly
Source: Rev Cardiovasc Med. 2025 Sep 28;26(9):38745. doi: 10.31083/RCM38745 (PMC12516742; doi:10.31083/RCM38745)
Supplement: Supplementary file 1 [file 2153-8174-26-9-38745-s1.docx]

**Supplementary Table 1.** Differences in GH-related hormones among the three abnormal groups.

|  | **Interventricular septal thickening (n=42)** | **left atrial enlargement (n=21)** | **abnormal E/A ratio**  **(n=51)** | ***F/H*** | ***P*** |
| --- | --- | --- | --- | --- | --- |
| OGTT-GH(µg/L) | 11.15(5.71,23.43) | 18.2(8.08,65.74) | 13.2(6.75,47.90) | 3.043 | 0.218 |
| GH(µg/L) | 15.8(9.47,29.58) | 23.0(10.35,83.5) | 21.0(10.2,35.30) | 1.222 | 0.543 |
| IGF-1(µg/L) | 750.05±246.12 | 788.33±228.57 | 733.84±210.69 | 0.426 | 0.654 |
| IGF-1/ULN | 3.04±0.98 | 3.33±1.00 | 3.06±0.91 | 0.748 | 0.476 |

Note: Data are expressed as mean ± standard deviation or *M(P_25_, P_75_)*. P values are from ANOVA or Kruskal-Wallis *H* test.

**Supplementary Table 2** Cutoff values of IGF-1/ULN generated by ROC curve analysis.

|  | **AUC** | **95%CI** | **cut-off** | **Sensitivity (%)** | **Specificity (%)** | ***P*** |
| --- | --- | --- | --- | --- | --- | --- |
| Interventricular septal thickening | 0.628 | 0.324-0.536 | 3.43 | 38.1 | 88 | 0.023* |
| left atrial enlargement | 0.701 | 0.575-0.826 | 3.03 | 66.7 | 69.8 | 0.004** |
| abnormal E/A ratio | 0.653 | 0.555-0.753 | 2.49 | 74.5 | 54.5 | 0.004** |

Notes: AUC: the area under the receiver operating characteristic curve, CI: confidence interval.

**P*<0.05, ***P*<0.01.
